# The three-dimensional conformation and activity of mitochondria in syncytial male germ line-cysts of medicinal leeches

**DOI:** 10.1007/s00441-023-03825-y

**Published:** 2023-08-29

**Authors:** Natalia Diak, Małgorzata Alicja Śliwińska, Sebastian Student, Piotr Świątek

**Affiliations:** 1 University of Silesia in Katowice, Faculty of Natural Sciences, Institute of Biology, Biotechnology and Environmental Protection, Bankowa 9, 40-007 Katowice, Poland; 2 Department of Medical Genetics, Faculty of Medical Sciences in Katowice, Medical University of Silesia, Katowice, Poland; 3 Laboratory of Imaging Tissue Structure and Function, Nencki Institute of Experimental Biology, Polish Academy of Sciences, Ludwika Pasteura 3, 02-093 Warsaw, Poland; 4 Silesian University of Technology, Faculty of Automatic Control, Electronics and Computer Science, Akademicka 16, 44-100 Gliwice, Poland; 5 Silesian University of Technology, Biotechnology Center, Krzywoustego 8, 44-100 Gliwice, Poland

**Keywords:** SBEM, Fusion, Fission, Hirudinidae, Mitochondrial membrane potential

## Abstract

We studied the spatial conformation and activity of mitochondria in the developing syncytial male germline cysts during spermatogenesis of the medicinal leeches using light, fluorescent, transmission electron microscopy, and serial block-face scanning electron microscopy. In cysts with spermatogonia and spermatocytes, mitochondria form networks and are in a dynamic hyperfusion state, while in cysts with spermatids, a single huge mitochondrion is observed. As spermiogenesis progresses, this huge mitochondrion is finally located in the future midpiece. The highest activity, in terms of membrane potential, of the mitochondria in *H. medicinalis* germline cysts was observed in cysts with spermatocytes; the lowest was in cysts with late elongated spermatids.

**Supplementary Information:**

The online version contains supplementary material available at 10.1007/s00441-023-03825-y.

## Introduction

Syncytial germline cysts are formed in animals during early gametogenesis as a result of cell divisions that are followed by incomplete cytokinesis (Robinson and Cooley 1996; Pepling et al. 1999; Świątek et al. 2009, 2014; Lu et al. 2017; Świątek and Urbisz 2019; Chaigne and Brunet 2022). During spermatogenesis, the formation of germline cysts is a conserved aspect of sperm formation (Yoshida 2016). Male germ cells are interconnected inside cysts until the final separation during spermation (Greenbaum et al. 2011; Yoshida 2016). Such connections consist in specific cellular junctions called intercellular bridges, cytoplasmic bridges, or ring canals (Greenbaum et al. 2011; Haglund et al. 2011; Yoshida 2016, Lu et al. 2017). These intercellular bridges are modified contractile rings that do not close during late cytokinesis (Chaigne and Brunet 2022). Consequently, the cytoplasm and cell constituents such as organelles and macromolecules can be shared between interconnected cells, and such cysts are a kind of syncytium (Greenbaum et al. 2011; Haglund et al. 2011; Chaigne and Brunet 2022). Intercellular bridges are wide cytoplasmic channels (0.2 to 15 μm in diameter) (Greenbaum et al. 2011; Haglund et al. 2011; Świątek et al. 2014). Due to the number of intercellular bridges that a given cell has and the spatial distribution of the germ cells within a cyst, several types of male cyst organization (architecture) have been recognized: (a) linear, where cells form chains, and each one has two bridges, except for the terminal ones (e.g., in mammals); (b) branched, where cells bear as many brides as the times they divided (e.g., in certain insects including *Drosophila melanogaster*) and (c) cysts equipped with a spherical anuclear central mass of cytoplasm where each cell has one bridge connecting it to the central cytoplasm (e.g., annelids) (de Cuevas et al. 1997; Pepling et al. 1999; Matova and Cooley 2001; Świątek et al. 2009; Świątek and Urbisz 2019; Chaigne and Brunet 2022). In leeches, the central cytoplasmic mass in the male germline cyst is termed cytophore (Jamieson 2006). The cytophore interconnects all the germ cells of a cyst and mediates cytoplasm and macromolecules sharing (Ventelä, 2006; Świątek et al. 2009; Ben Ahmed et al. 2015; Małota and Świątek 2016; Yoshida 2016; Świątek and Urbisz 2019). The cytophore is formed during cyst formation as a result of the centripetal transport of cytoplasm from cells towards the centre of the cyst (Świątek et al. 2009; Świątek and Urbisz 2019).

Mitochondria are cellular organelles that are surrounded by two membranes (inner and outer mitochondrial membrane) and contain their own DNA (mtDNA) (Cherry et al. 2016). Mitochondria perform multiple functions, and the most famous is energy production by generating most of the adenosine triphosphates (ATP) (Alberts et al. 2005; Friedman and Nunnari 2014; Hoitzing et al. 2015). Mitochondria are also responsible for stress reactions (Shaughnessy et al. 2014), apoptosis signaling (Chan 2006a), or cell cycle regulation (Finkel and Hwang 2009; Mitra et al. 2009). Mitochondrial morphology and dynamics have been analyzed numerous times, also in medical studies, because their functioning is connected with numerous diseases such as, e.g., Parkinson's, cancer and Alzheimer's (Hoitzing et al. 2015). Mitochondria also play an essential role during gametogenesis.

Mitochondrial dynamics is regulated by two opposite processes, i.e., fusion and fission (Twig et al. 2008). These processes play a role in cell survival and adaptation to changing conditions required for their growth and functioning. Fusion and fission are also necessary to distribute mitochondria during cell differentiation properly (Twig et al. 2008). During fusion, mitochondria form a network or networks (Cherry et al. 2016). Mitochondrial membranes of neighboring mitochondria merge, and their matrixes are mixed (Chan 2012). Several possible functions for mitochondrial networks have been proposed, e.g., the degradation of dysfunctional mitochondria or mtDNA complementation (Hoitzing et al. 2015). The second process (fission) is the opposite; during fission, individual mitochondria are separated from the network (Cherry et al. 2016). Hoitzing et al. (2015), based on the ratio between fusion and fission, distinguished four states of mitochondria dynamics (conformation): microfusion (when fusion is rare, most mitochondria are individual); mesofusion (fusion is rare, fission dominates); dynamic hyperfusion (fusion dominates, individual mitochondria are rare); and static hyperfusion (when fission is rare and most of the mitochondria are united into a three-dimensional network). Mechanisms that regulate mitochondrial quality control are, e.g., fission, fusion, and mitophagy (i.e., the degradation of mitochondria via autophagy), which is necessary for maintaining a healthy mitochondrial population (Hoitzing et al. 2015).

Analyses of mitochondrial parameters have generally been performed on model organisms such as mammals or yeast (Rafelski 2013; Varuzhanyan and Chan 2020). However, studies that present the 3D morphology of mitochondria at the ultrastructural level are rare (Leapman et al. 2016; Tworzydlo et al. 2016; Urbisz et al. 2020; Urbisz et al. 2022; Hayes et al. 2021; Leung et al. 2021). Thus, the knowledge about the spatial organization of mitochondria is still limited. Mitochondria are particularly interesting in studying gametogenesis: in oogenesis, because they are transmitted to the next generation via the female line; in spermatogenesis because they are responsible for sperm motility (Ankel-Simons and Cummins 1996; Ramalho-Santos 2011; Gilbert and Barresi 2016). Because in animals, the offspring generally inherit only maternal mitochondria (with the unique known exception in bivalve mollusks with Doubly Uniparental Inheritance of mitochondria: Breton et al. 2007; Zouros 2013; Ladoukakis and Zouros 2017), numerous studies investigated the behavior of mitochondria during oogenesis. These studies focused mainly on the function of mitochondria in the female germ cells, their segregation, and their quality (e.g., recently reviewed in Bilinski et al. 2017; Tworzydlo et al. 2020). Paternal mitochondria usually do not enter the oocyte, and when they enter the ooplasm, they are eliminated quickly (Ankel-Simons and Cummins 1996; Gilbert and Barresi 2016). Although male mitochondria usually do not provide paternal genetic information to the embryo, it is well known that they are responsible for sperm movement, which is directly connected with the ability of sperm to fertilize an egg cell and sperm quality (Ankel-Simons and Cummins 1996; Ramalho-Santos 2011; Gilbert and Barresi 2016). To date, numerous microscopic techniques have been used to analyze the functioning of the mitochondria in germline cells, including confocal laser scanning microscopy and flow cytometry (e.g., Gravance et al. 2000; Marchetti et al. 2002). The mitochondrial membrane potential (MMP) is one of the parameters that are easy to measure in living cells using, e.g., JC-1 staining, and MMP reflects the level of ATP production (Amaral et al. 2013; Milani and Ghiselli 2015; Milani and Ghiselli 2015a, b).

Spermatogenesis in leeches (like in other clitellate annelids) seems to be an excellent model for studying the morphology and functioning of mitochondria in germ cells. Indeed, from early generations of spermatogonia to the formation of the mature sperm, the somatic cells do not envelop the germ cells, and male cysts float freely within the coelomic fluid of the testes (Jamieson 2006).

Previous studies on spermatogenesis in leeches mainly focused on spermiogenesis (e.g., Pastisson 1965, 1975, 1977; Lora Lamia Donin and Lanzavecchia 1974; Garavaglia et al. 1974; Wissocq and Malécha 1975), that is the differentiation from spermatids to mature spermatozoa. Consequently, the ultrastructural details of sperm formation and sperm ultrastructure are well-known (reviewed in Jamieson 1981, 2006; Ferraguti 1983, 2000), including in medicinal leeches (e.g., Wissocq and Malécha 1975; Ben Ahmed et al. 2015). Our idea was to analyze conformation and activity of mitochondria throughout the whole spermatogenesis, from spermatogonia to late spermiogenesis. We used the fluorescent dyes MitoTracker Orange to detect mitochondria and JC-1 to assess their activity in not fixed (live) cysts. Light and transmission electron microscopy were used to describe the ultrastructure. Moreover, we employed serial block-face scanning electron microscopy (SBEM) to obtain the three-dimensional reconstructions of germline cysts and visualize the spatial conformation of mitochondria during the consecutive stages of spermatogenesis.

## Materials and methods

### Animal samples

The European medicinal leeches *Hirudo medicinalis, H. orientalis* and *H. verbana* were purchased from a commercial leech bio-farm ‘Bio-Gen’ in Namysłów (southern Poland). Specimens of *H. nipponia* were collected on Honshu Island, Japan. Because *H. medicinalis* is partially protected by Polish law, the research team obtained the appropriate permission from the Regional Director for Environmental Protection in Katowice (permission number WPN.6401.239.2017.MS). Leeches were kept in aquaria at a temperature of 20 °C and a lighting regime 12 h of light and 12 h of darkness.

### Light microscopy and transmission electron microscopy

These methods were used for four species of leeches of the genus *Hirudo*, i.e., *H. medicinalis*, *H. orientalis*, *H. verbana* and *H. nipponia*. We analyzed five individuals of the species: *H. medicinalis, H. orientalis*, *H. verbana* and two individuals of *H. nipponia*. Leech specimens were narcotized with 50% ethanol for 1–2 min and sectioned. Dissected testes were fixed in 2.5% glutaraldehyde in a phosphate buffer for 24 h at room temperature. After fixation, the material was rinsed several times with a mixture of a 50-ml 0.1 M phosphate buffer (pH = 7.4) and 50 ml of ddH_2_O to which 4.6 g of saccharose were added. Afterwards, the material was postfixed for 2 h in a mixture of 1% OsO_4_ in a 0.1-M phosphate buffer (pH = 7.4), dehydrated in a graded series of ethanol, which was replaced by acetone, and then embedded in an Epoxy Embedding Medium Kit (Sigma, St. Louis, MO). Semi-thin sections (0.7 μm thick) were cut on a LEICA Ultracut ultramicrotome. The semi-thin sections were stained with 1% methylene blue in 0.5% borax. The semi-thin sections were analyzed using an Olympus BX60 light microscope equipped with an Olympus XC50 digital camera and CellSens Standard software. The ultra-thin sections were contrasted with uranyl acetate (30 min) and lead citrate (20 min) and were examined using a Hitachi H500 transmission electron microscope.

### Three-dimensional reconstructions–Serial Block Face Scanning Microscopy

This method was only used for *H. medicinalis* (two individuals). Tissue fixation for SBEM was done according to a modified protocol of Deerinck et al. (2010), ver. 7-01-2010. The dissected testes were fixed in 2.5% glutaraldehyde in a 0.1-M phosphate buffer (pH 7.4) at room temperature for 2 h. After washing in the same buffer, the samples were post-fixed for 1 h with a mixture of 3% potassium ferrocyanide in a 0.3-M cacodylate buffer and an equal volume of a 4% aqueous solution of osmium tetroxide. The tissue was then washed three times for 5 min in ddH_2_O and incubated for 20 min in a 1% aqueous solution of thiocarbohydrazide (Ted Pella) at 60 °C. After that, the samples were washed three times for 5 min in ddH2O and placed in 2% aqueous osmium tetroxide for 30 min; then, the tissue was rewashed three times for 5 min in ddH_2_O and was incubated overnight in 1% aqueous uranyl acetate at 4 °C. The samples were then rinsed three times for 5 min in ddH_2_O and put into a freshly prepared Walton’s 0.66% lead aspartate for 30 min at 60 °C. Then, they were washed five times for 3 min in ddH_2_O and dehydrated for 10 min in a series of 30, 50, 70, and 96% ethanol solutions, placed in anhydrous 100% ethanol three times for 20 min, for 15 min in a 1:1 solution of acetone and ethanol, and twice for 15 min in 100% acetone. After dehydration, the samples were placed in a mixture of 50% Epoxy Embedding Medium (Sigma-Aldrich, St. Louis, MO, USA) in acetone for 3 h, then left overnight for the acetone to evaporate. The prepared material was embedded between two Aclar (EMS) layers and left to polymerize. For the SBEM technique, a small piece of tissue was cut out with a razor blade and was attached to an aluminum pin (metal rivets, Oxford Instruments) with a minimal amount of cyanoacrylate glue. The excess of resin was trimmed on each side of the sample to minimize charging. Next, samples were attached with silver paint (Ted Pella, 16062-15) to the pin and dried for 24 h. Stacks of images from the serial 100–150 nm ultrathin sections were collected using a scanning electron microscope Sigma VP (Zeiss) equipped with an ultramicrotome chamber 3View (Gatan, Pleasanton, CA, USA) and Digital Micrograph software (Gatan) and a backscattered electron detector. Pixel size for spermatogonia and late elongate spermatids was 13.8 nm; for spermatocytes and isodiametric spermatids was 15.2 nm, and for early elongate spermatids was 12.5 nm. This research was carried out in the Laboratory of Imaging Tissue Structure and Function, Warsaw (Nencki Institute of Experimental Biology). The free software ImageJ (Schindelin et al. 2012) was used to prepare the reconstructions. TrakEM2 was used to create the 3D reconstructions. TrakEM2 is a special plugin in ImageJ that can be used for, e.g., dimensional modeling and image stitching and editing. The mitochondria and germ cyst were manually contoured, and then 3D models were automatically generated. The movies were saved in the AVI format. All mitochondria were also segmented using surface modeling available in Imaris using a manual outline. For each segmented cell, the volume was determined using the statistics generator available in Imaris.

Before analyzing the material with the SBEM method, the same samples were analyzed at transmission electron microscopy and bright field microscopy to check the fixation quality. The pins with attached embedded tissue were mounted in a Leica UCT ultramicrotome. Semi-thin and ultra-thin sections were examined as described in the previous section.

### Detecting the mitochondrial membrane potential (MMP) level–fluorescent microscopy and statistical analysis

MitoTracker Orange CMTMRos (M7510; Life Technologies), and JC-1 fluorochrome (5,5′,6,6′-tetrachloro-1,1′,3,3′-tetraethylbenzimidazolyl carbocyanine iodide) (T3168; Life Technologies) were used to visualize in living cysts the mitochondria and Hoechst 33342 (H1399; Life Technologies) for DNA. MitoTracker Orange was used to visualize the exact localization of the mitochondria in cell cysts. JC-1 was used to mark mitochondrial activity (by detecting the mitochondrial membrane potential) by distinguishing active and inactive mitochondria. Within mitochondria with low MMP potential (inactive mitochondria), JC-1 has a monomeric form and gives green fluorescence; when the mitochondrial membrane becomes polarized (active mitochondria), JC-1 aggregates (form J-aggregate) and gives red fluorescence. This method was only used for *H. medicinalis*; 10 individuals were taken for analysis. The dissected testes were transferred to a cell culture medium (Dulbecco’s phosphate buffered saline, DPBS). The testes were then fragmented manually under a stereomicroscope to obtain the cyst suspension in DPBS. For each staining, live-cell dyes were diluted in DPBS at a ratio of 1:1000; the labeling time varied between 10 and 20 min. After staining, the cyst suspension was immediately placed onto microscopic slides using a glass pipette. The slides were examined under an Olympus FV1000 confocal laser scanning microscope.

Based on the results from the confocal microscopy, statistical analysis was performed. The statistical analysis calculated the mean percentage of activated and non-activated mitochondrial spots in the germ-cell cysts and the cytophore. The cysts were segmented first using the surface modeling available in the ImageJ using a manual outline. For each segmented cell and cytophore, the signals from JC-1 were detected using both summarized channels (sum of intensity signals indicated by a green and red fluorescence obtained by microscopic analysis), excited with 488 nm and 568 nm lasers. The spots were detected using image segmentation with the region-growing method. An arbitrary mean intensity threshold for the summarized dye signals was used to detect the number of mitochondrial spots (Mancas et al. 2005). In all samples, the aggregate dye excited at 568 nm red channel (JC-1 activated-mitochondria with high membrane potential) was used to determine the number of activated mitochondrial spots. The aggregate dye excited at 488 nm green channel (JC-1 inactivated-mitochondria with low membrane potential) was used to determine the number of inactivated mitochondrial spots. As a result, we calculated the mean percentage of activated and inactivated mitochondrial spots in each analyzed germ-cell cyst. We used the Wilcoxon test comparisons between group complex levels (i.e., spermatogonia, spermatocytes, isodiametric spermatids, early elongate spermatids, and late elongate spermatids) for the statistical analysis.

## Results

Since no histological and ultrastructural differences between the species were observed, the descriptions presented below are common for all the species studied. The analysis of MMP potential was done only in *H. medicinalis.*

### General cyst organization

**Fig. 1:**
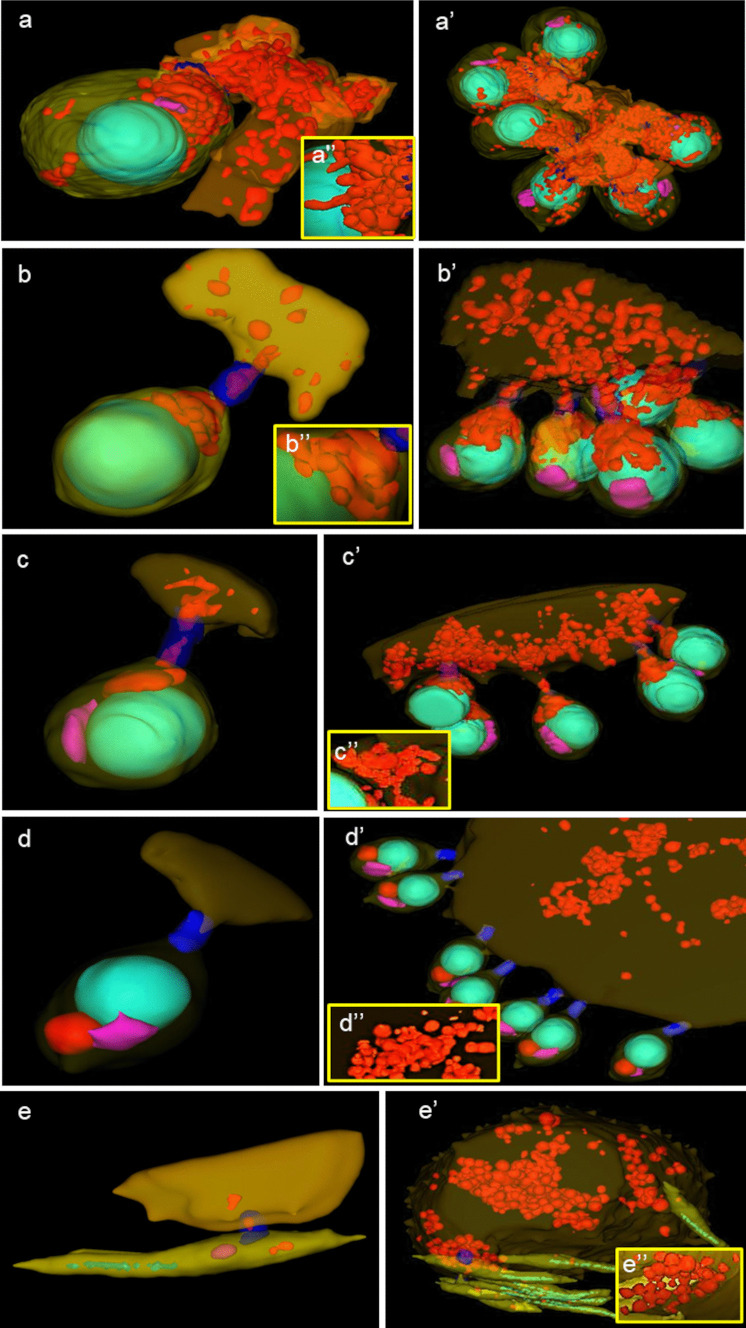
**a–e** Single images from three-dimensional reconstruction of germline cells and a’-e’ fragment of germline cyst in the consecutive stages of spematogenesis of *Hirudo Medicinalis*. **a**, **a’**, **a’’** Spermatogonia, **b**, **b’**, **b’’** spermatocytes, **c**, **c’**, **c’’** isodiametric spermatids, **d**, **d’**, **d’’** early elongate spermatids, **e**, **e’**, **e’’** late elongate spermatids. Yellow—cells; orange—cytophore fragment; light green—nuclei; red—mitochondria; pink—Golgi complex; blue—intercellular bridge. Serial block face scanning microscopy (SBEM). **a’’–e’’**—higher magnification of the mitochondrial network. In panels a-c networks from germ cells, in **d–e** networks from cytophores

**Fig. 2:**
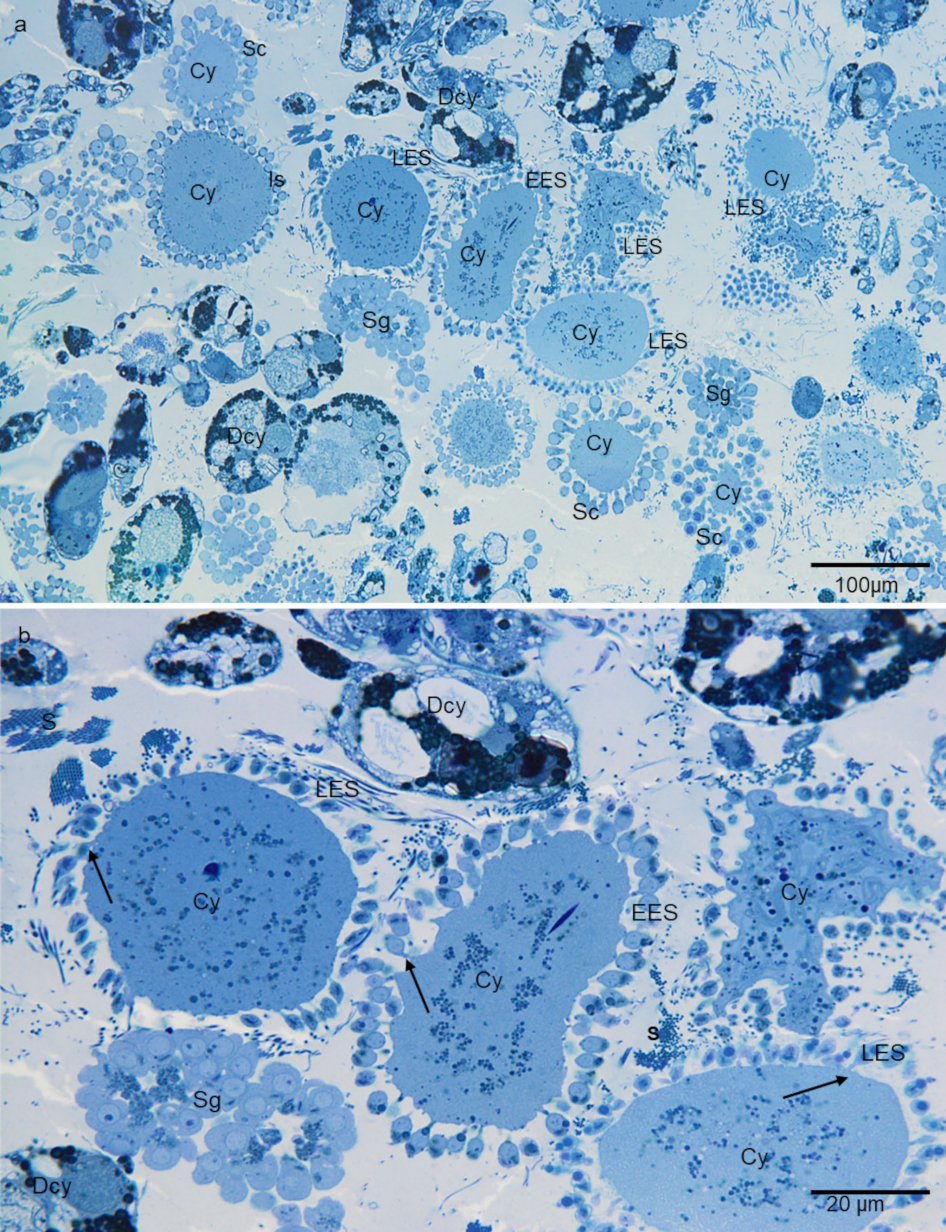
**a**, **b** Germline cysts inside the testisac of *H. medicinalis*. Sg—spermatogonia, Sc—spermatocytes, Is—isodiametric spermatids, EES—early elongate spermatids, LES—late elongate spermatids, Cy—cytophore, Dcy—degenerating cytophore, S—sperm, arrow—intercellular bridges. Light microscopy (LM), epon semi-thin sections stained with methylene blue

**Fig. 3:**
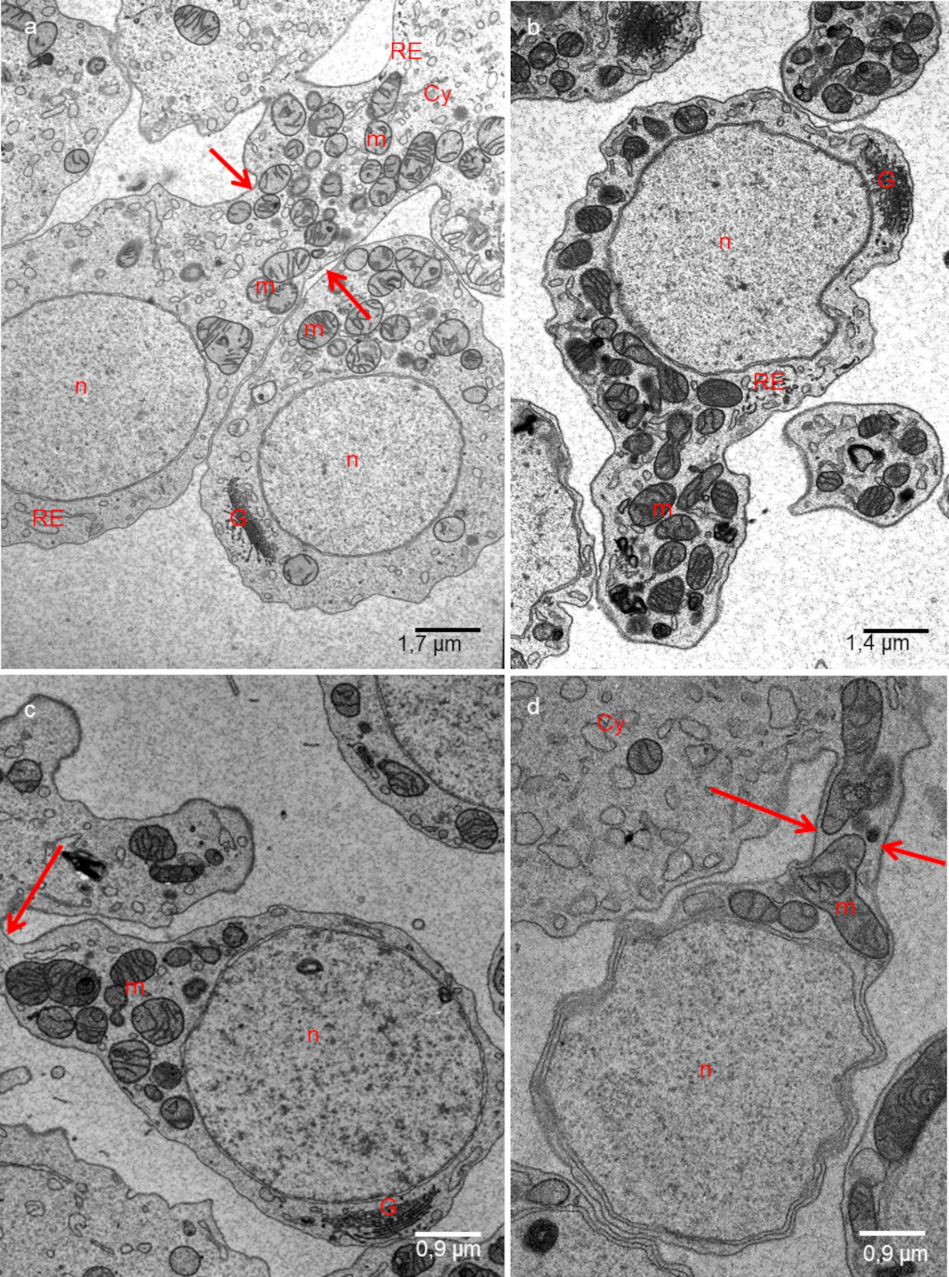
**a**, **b** Ultrastructure of spermatogonia. **c**, **d** Ultrastructure of spermatocytes. **a**, **d** *H. medicinalis*. **b** *H. verbana*; **c** *H. nipponia*. m—mitochondria; n—nucleus; G—Golgi complex, Cy—cytophore; RE—endoplasmic reticulum, arrows—intercellular bridge. **a** Classic fixation, **b–d** SBEM fixation. Transmission electron microscopy (TEM)

**Fig. 4:**
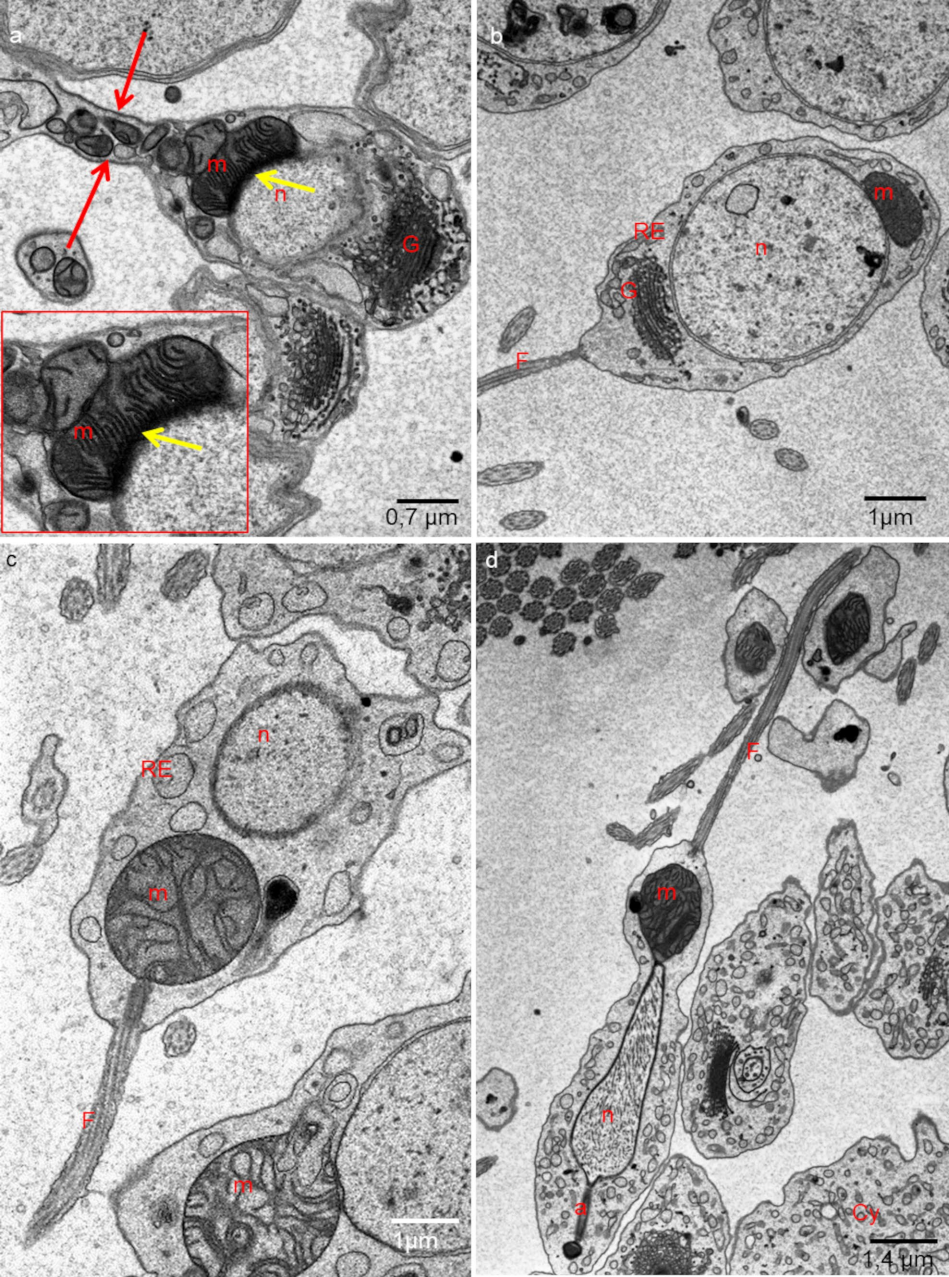
**a**, **b** Ultrastructure of isodiametric spermatids. **c**, **d** Ultrastructure of early elongate spermatids. **a**, **c** *H. medicinalis*. **b**, **d** *H. nipponia*. a—acrosome, m—mitochondria; n—nucleus; G—Golgi complex, Cy—cytophore; RE—endoplasmic reticulum, F—flagellum, yellow arrow—electron-dense material, red arrows—intercellular bridge. SBEM fixation, TEM

**Fig. 5:**
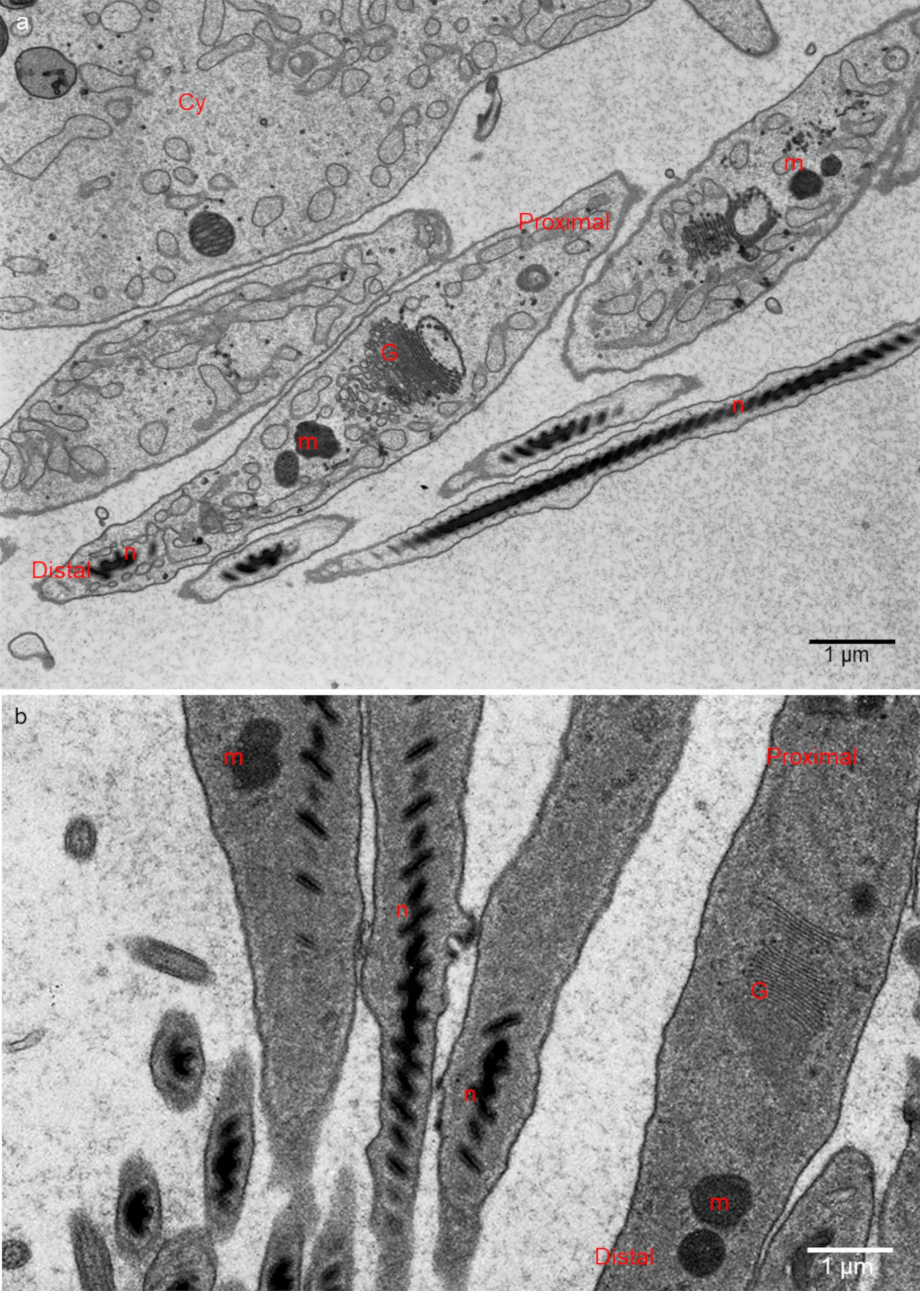
**a**, **b** Ultrastructure of late elongate spermatids. **a** *H. nipponia*. **b** *H. medicinalis*. m—mitochondria; n—nucleus; G—Golgi complex, Cy—cytophore; RE—endoplasmic reticulum. Classic fixation, TEM

**Fig. 6:**
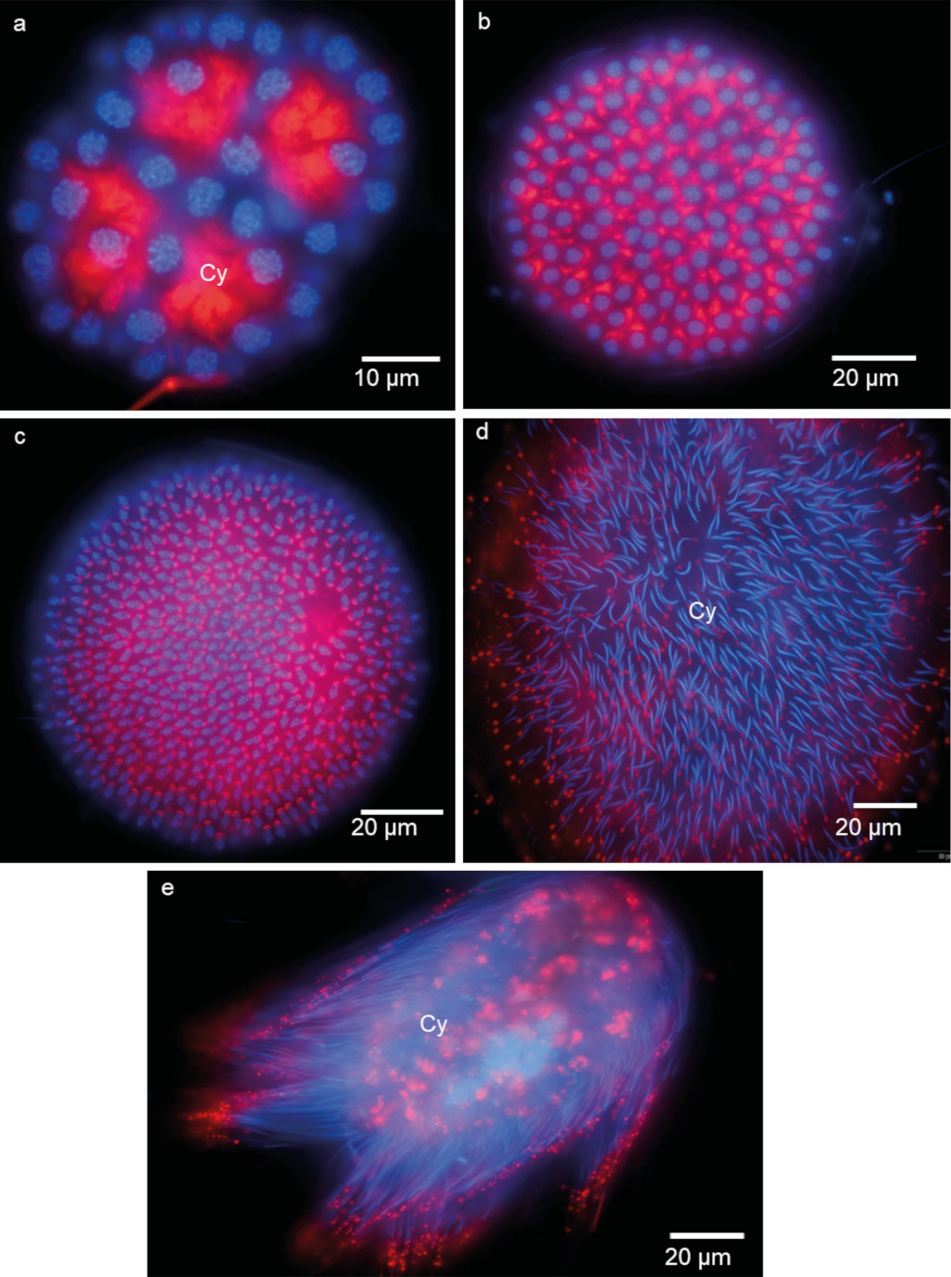
Localization of mitochondria in the consecutive stages of gem-line cyst development of *H. medicinalis*. **a** A cyst with spermatogonia, **b** a cyst with spermatocytes, **c** a cyst with isodiametric spermatids, **d** a cyst with early elongate spermatids, **e** a cyst with late elongate spermatids. Cy—cytophore. Fluorescence microscopy (FM), Live staining—red signals mark mitochondria visualized by MitoTracker labeling, blue signals mark chromatin visualized by Hoechst 33,342 labeling

**Fig. 7:**
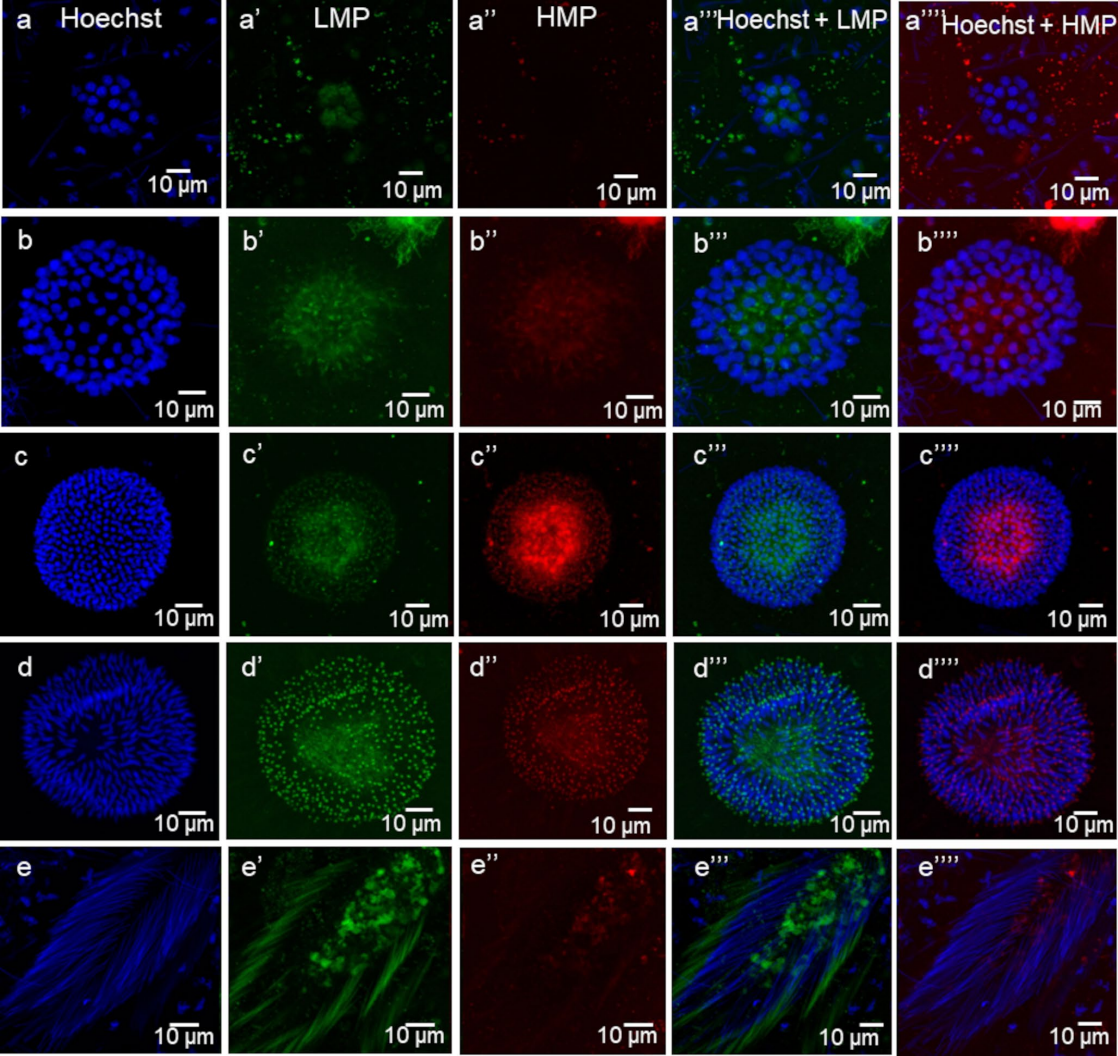
The mitochondria membrane potential (MMP) activity in the consecutive stages of cyst development visualized by JC-1 staining of *H.medicinalis*. Green signal—mitochondria with a low MMP, red signal—mitochondria with a high MMP, blue signal—cell nuclei visualized by Hoechst 33,342 staining. **a’**–**a’’’’** A cyst with spermatogonia, **b–b’’’’** a cyst with spermatocytes, **c–c’’’’** a cyst with isodiametric spermatids, **d–d’’’’** a cyst with early elongate spermatids, **e–e’’’’** a cyst with late elongate spermatids. FM

The leech male germ cells inside the testis are united into syncytial groups of cells known as cysts, clones, or clusters (Figs. 1, 2, 3, 4, 5, 6, 7). Each male germline cyst has a cytophore that occupies the cyst center, whereas cells are arranged peripherally (Figs. 1a–e, a’–e’ and 2). Each germ cell is connected to the cytophore by a single intercellular bridge (IB) (Figs. 1a–e, a’–e’ and 2b). Although the cysts are in different stages of development (thus, there is no synchrony between the cysts), all the germ cells in a given cyst are at the same developmental stage (synchronous development of all the cells that are interconnected in a given cyst). Therefore, individual cysts uniting spermatogonia (Figs. 1a–a’’ and 2), spermatocytes (Figs. 1b–b’’ and 2a), or spermatids (isodiametric, early, and late elongate spermatids) (Figs. 1c–e’’ and 2) occur in each testis. The terminology of spermatogenesis stages was adopted after Jamieson (1981). Based on photos from the confocal microscope, the number of cells per cyst with isodiametric spermatids was counted. The number of cells in such cysts varies slightly and ranges from 507 to 516. The mean number of cells in the cyst was 512 (*N* = 10). No germ cell divisions were observed during the studies. Assuming that the divisions within a given cyst are synchronous, as was observed multiple times during spermatogenesis in other annelids (e.g., Adiyodi and Adiyodi 1983; Olive 1983; Jamieson 2006; Świątek et al. 2009; Małota and Świątek 2016; Ben Ahmed et al. 2019) it was calculated that clones with 507–516 spermatids were formed as a consequence of nine synchronic divisions (2^9^ = 512). On this basis, the number of cells in a given cyst in the remaining stages was estimated, making it possible to distinguish successive stages. It was shown that spermatogonia divide seven times to give 128 primary spermatocytes. They enter meiosis and divide to give 256 secondary spermatocytes, and after the second meiotic division, the cyst ultimately consists of 512 spermatids.

### Spermatogonia

**Fig. 8:**
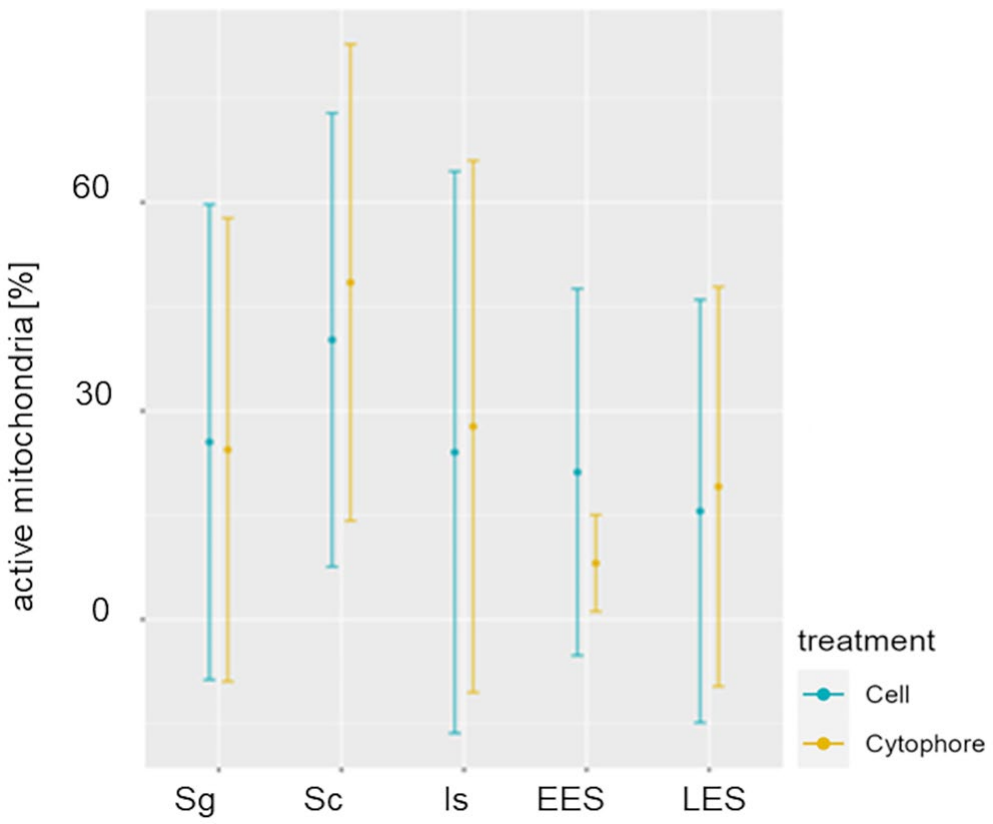
Means and standard deviations of mitochondria with the high membrane potential (active mitochondria) in cells (blue) and cytophore (yellow) during the successive stages of spermatogenesis. Sg—spermatogonia, Sc—spermatocytes, Is—isodiametric spermatids, EES –early elongate spermatids, LES—late elongate spermatids

**Fig. 9:**
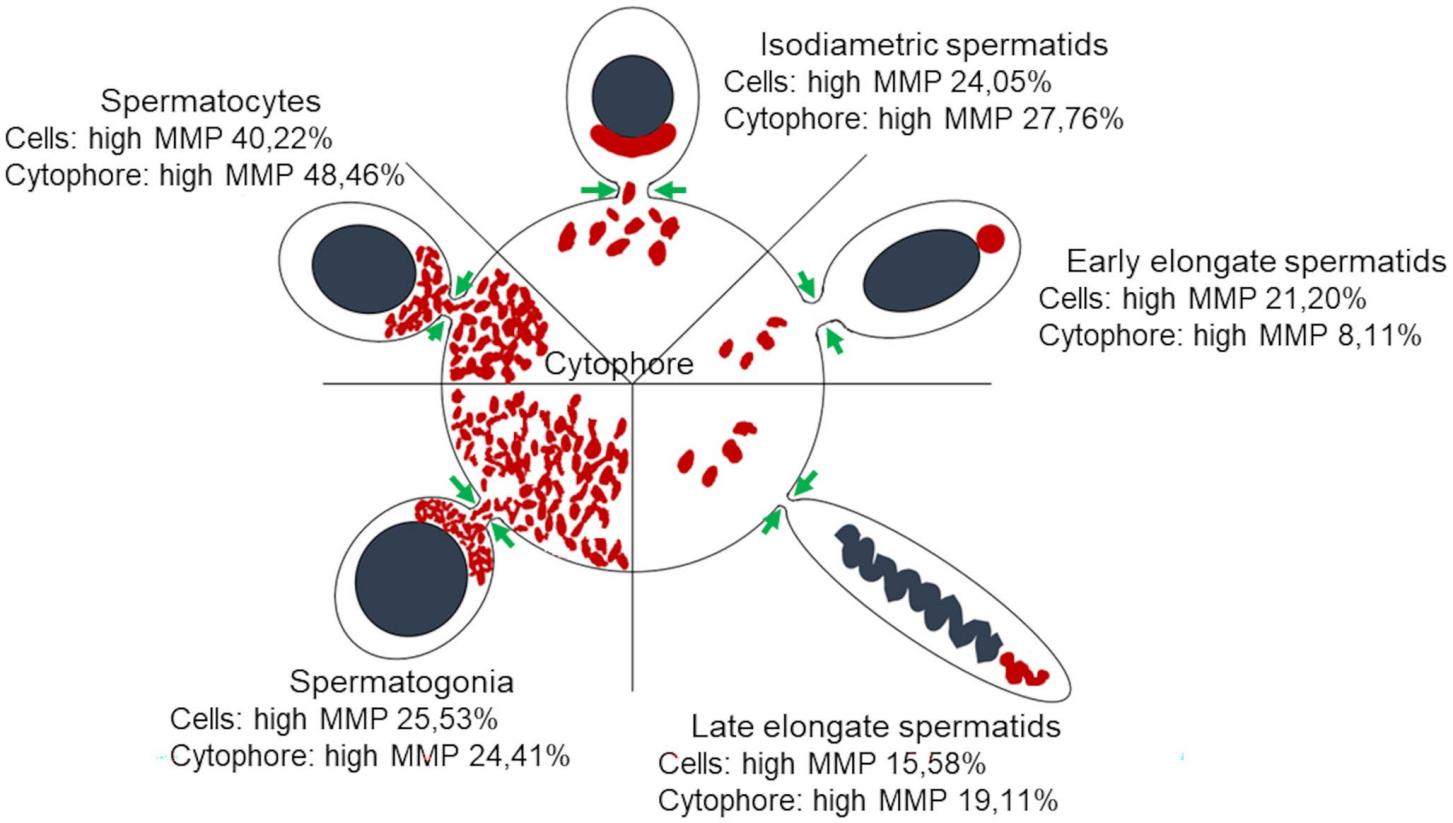
A scheme of mitochondria (red) distribution in the consecutive stages of spermatogenesis. Grey—cell nuclei, green arrows—intercellular bridges. The cell and cytophore proportions do not reflect the real size

Spermatogonia are pear-shaped cells (Figs. 1a–a’, 2a, b and 3a, b; Videos S1a and S1b) with a large nucleus that occupies most of the cell volume (Fig. 3a, b). In these cells, most mitochondria are located in the proximal part of the cells (i.e., cell pole with the IB) and are oval or club-shaped (Figs. 1a–a’, 3a, b, 6a and 7a’–a’’’’). In spermatogonia, numerous mitochondria were connected and formed a network (Fig. 1a–a’’; Videos S1a and S1b). The average volume of individual networks is about 16.25 µm^3^, of the single mitochondrion is about 0.12 µm^3^. Some mitochondria were found within the IBs and cytophore (Figs. 1a–a’ and 3a; Videos S1a and S1b). All these mitochondria had well-developed cristae, which are more or less parallel between each other and perpendicular to the long mitochondrial axis (Fig. 3a, b). The mitochondrial matrix was electron-dense (Fig. 3a, b). The differences in the density of the mitochondrial matrix among figure panels are due to the fixation method (see details in sections “Light microscopy and transmission electron microscopy” and “Three-dimensional reconstructions–Serial Block Face Scanning Microscopy” in “Material and methods”). JC-1 staining showed that in cysts with spermatogonia, the average number of mitochondria with high MMP was 25.53% in cells and 24.41% in cytophore (Table 1; Figs. 8 and 9).

**Table 1:** The ratio of active and inactive mitochondria in different cyst compartments. Percentage of mitochondria with low and high membrane potential in different cluster compartments during spermatogenesis

	Cells		Cytophore	
	High MMP	Low MMP	High MMP	Low MMP
Spermatogonia	25.53%	74.47%	24.41%	75.59%
Spermatocytes	40.22%	59.78%	48.46%	51.54%
Isodiametric spermatids	24.05%	75.95%	27.76%	72.24%
Early elongate spermatids	21.20%	78.80%	8.11%	91.89%
Late elongate spermatids	15.58%	84.42%	19.11%	80.89%

### Spermatocytes

In spermatocytic cysts, the organelle composition, distribution, and ultrastructure are the same as in spermatogonia (Figs. 1b–b’’and 3c, d; Videos S2a and S2b). However, they differ in the volume. The a,verage volume of individual networks is about 3.57 µm^3^, of the single mitochondrion is about 0.07 µm^3^. Mitochondria also form a network and are located almost exclusively in the proximal part of the cells (Figs. 1b–b’’; 3c, d, 6b and 7b’–b’’’’; Videos S2a and S2b). The general ultrastructure of mitochondria did not change compared to the previous stage (Fig. 3c, d). JC-1 staining revealed that the average number of mitochondria with high MMP in spermatocytic cysts was 40.22% in cells and 48.46% in cytophore (Table 1; Figs. 8 and 9).

### Spermatids

Based on the analysis of the shape and distribution of organelles during spermiogenesis, three types of spermatids have been distinguished: isodiametric spermatids (Figs. 1c–c’’, 2a and 4a, b; Videos S3a and S3b), early elongate spermatids (Figs. 1d–d’’, 2b and 4c, d; Videos S4a and S4b) and late elongate spermatids (Figs. 1e–e’’, 2b and 5a, b; Videos S5a and S5b). Isodiametric spermatids have a similar size on each axis and a rounded shape (Figs. 1c–c’ and 4a, b; Videos S3a and S3b), which elongates in the following stages (Figs. 1d–d’, e–e’, 4c, d and 5a, b; Videos S4a and S4b to Videos S5a and S5b) Isodiametric spermatid is characterized by a large, spherical nucleus that occupies most of the cell volume (Figs. 1c–c’ and 4a, b; Videos S3a and S3b). In contrast to spermatogonia and spermatocytes, where numerous fused mitochondria are observed, a single crescent-shaped mitochondrion located at the proximal pole of the cell is characteristic of isodiametric spermatids (Figs. 1c–c’, 4a, b, 6c and 7c’–c’’’’; Videos S3a and S3b). However, we also observed several/groups of small mitochondria in the proximal pole of the cell and inside the IBs, which were in fusion (Figs. 1c–c’’ and 4a, b; Videos S3a and S3b).

The average length (along the long axis) of crescent-like mitochondria is 2.08 µm, while the width is 0.68 µm (*N* = 10). The average volume of this mitochondrion is 2.34 µm^3.^ These crescent-like mitochondria are located in close proximity to cell nucleus (Figs. 1c–c’ and 4a, b; Videos S3a and S3b). The nuclear envelope in the contact zone with mitochondrion is electron-dense (Fig. 4 an inset). Crescent-like mitochondria have an electron–dense matrix and well-developed cristae running perpendicular to their long axis (Fig. 4a, b). In the case of cysts with isodiametric spermatids, mitochondria were also observed in the IBs and the cytophore (Figs. 1c–c’ and 4a). The general ultrastructure of these mitochondria did not change in comparison to the cells in the previous stage (Figs. 1c–c’’ and 4a). In cysts with isodiametric spermatids, the average number of mitochondria with high MMP was 24.05% in cells and 27.76% in cytophore (Table 1; Figs. 8 and 9).

In the next step of spermiogenesis, spermatids undergo further transformations, and the shape and arrangement of organelles change (Figs. 1d–d’’ and 4c, d; Videos S4a and S4b). Early elongate spermatids are characterized by elongated condensing nuclei (Figs. 1d–d’ and 4c, d; Videos S4a and S4b). The single mitochondrion became spherical and changed its position; now, it occupies the distal portion of the cell between the nucleus and the axoneme (Figs. 1d–d’, 4c, d, 6d and 7d’–d’’’’; Videos S4a and S4b). The average volume of this mitochondrion is 2.01 µm^3^. The mitochondrion also has an electron-dense matrix and well-developed cristae, which are oriented more or less parallel one to another (Fig. 4c, d). The change was found in the contact zone between nuclei and mitochondrion—in early elongated spermatids, no electron-dense material was observed between these two organelles (Fig. 4d). The average diameter of such single mitochondria is 1.48 µm (*N* = 10). In the case of cysts with early elongate spermatids, mitochondria were also observed in the cytophore and occasionally within IBs (Videos S4a and S4b). JC-1 staining showed that the average number of mitochondria with high MMP in cysts with early elongate spermatids was 21.20% in cells and 8.11% in the cytophore (Table 1). Late elongate spermatids is the last stage before mature sperm are formed and the last stage where the cells are interconnected into cysts (Figs. 1e–e’’, 5a, b, 6e and 7e–e’’’’; Videos S5a and S5b). Late elongate spermatid nuclei are elongated and twisted in a spiral (Figs. 1e–e’ and 5a, b; Videos S5a and S5b). The nucleus has the form of an electron-dense, spirally twisted structure with a characteristic shape resembling the letter “E” in cross-sections (Fig. 5a, b). From spermatogonia to early elongate spermatids Golgi apparatus is located in the distal part of the cell, more or less in the lateral position. In late elongate spermatids occur in the proximal part of cells. Fluorescence microscopy analyses revealed that mitochondrion was localized in the distal part of the cell (future midpiece) (Figs. 6e; and 7e’–e’’’’). The average volume of this mitochondrion is 0.11 µm^3^. In contrast to fluorescence and TEM analyses, in the cyst selected for three-dimensional reconstruction, we observed that the mitochondrion was localized in the proximal part of the cell, close to the Golgi apparatus (Fig. 1e–e’; Videos S5a and S5b). In cysts with late elongate spermatids, the average number of mitochondria with high MMP was 15.58% in cells and 19.11% in cytophore (Table 1). Summarized means for the activity of mitochondria, i.e., inactive mitochondria (mitochondria with low MMP) and active mitochondria (mitochondria with high MMP) in cells and cytophore during the successive stages of spermatogenesis, are shown in Figs. 8 and 9.

## Discussion

Spermatogenesis is a multi-stage process that leads to the formation of a motile male gamete, i.e., spermatozoon. During spermatogenesis, we can distinguish three main stages: spermatocytogenesis, spermatocytic stage (a meiotic phase), and spermiogenesis (Zhou and Griswold 2008; Gilbert and Barresi 2016). In leeches, all stages of spermatogenesis occur in the testes, where germ cells float freely within the coelomic fluid (Fernández et al. 1992). Spermiogenesis is a complicated cellular process in which a haploid germ cell (spermatid) transforms into a mature spermatozoon. During this process, the structure and shape of the spermatids change rapidly, i.e., the nucleus condenses and changes its shape; the acrosome and flagellum are formed; the cytoplasm is removed from the cells (the so-called residual body), and as a result, a specialized male gamete, a spermatozoon, is formed (Gilbert and Barresi 2016). In Hirudinida, the spermatozoon is an exceptionally complex gamete. It is composed of a complex acrosome (an anterior and posterior acrosome can be distinguished, the acrosome is always corkscrew-shaped and apparently prolongs the acrosome tube), a corkscrew-shaped nucleus, a midpiece (this structure is interposed between the nucleus and the flagellum and is usually composed of a single elongated and twisted mitochondrion), and a flagellum (for more detail see, e.g., Ferraguti 2000). The specific characters of the sperm ultrastructure in leeches, like in other clitellates, have been used to reconstruct the Clitellata phylogeny at different taxonomical levels, from species to families, for many years (the so-called spermiocladistics, see, e.g., Ferraguti and Erséus 1999; Jamieson 2006; Marotta et al. 2008; Marotta and Ferraguti 2009). Since most of the research has focused on spermiogenesis only, and there is little information about the earlier stages, we focused on mitochondria conformation and activity across spermatogenesis.

During spermatogenesis in clitellates, mitochondria change position within cells, as observed by, e.g., Jamieson 1981; Ferraguti 1983, 2000; Kalus 1993; Małota and Świątek 2016; Małota et al. 2019, and in this study for species of the genus *Hirudo*. The same distribution of these organelles was observed in the other medicinal leech species*–Hirudo troctina* (Ben Ahmed et al. 2015). In spermatogonia, spermatocytes, and isodiametric spermatids, fused mitochondria concentrate in the proximal pole of cells in the vicinity of the intercellular bridges (IBs). In contrast, in early and late elongated spermatids, they are located in the distal part of cells. Regarding the distribution of mitochondria during the consecutive stages of spermatogenesis, all techniques used to visualize mitochondria (see “Materials and methods” section) gave similar results, except for the location of mitochondria in late elongated spermatids. In these cells, fluorescence (MitoTracker and JC-1 labelings) and electron microscopy analyses revealed that a single mitochondrion was localized in the distal part of the cell. Unexpectedly, in the cyst selected for three-dimensional reconstruction (SBEM method), we observed that the mitochondrion was localized in the proximal part of the cell, close to the IB. It is difficult to interpret this phenomenon precisely—maybe the observed case of an atypical location of mitochondria in elongated spermatids is a slightly different timing in the differentiation process or a pathological situation resulting in the formation of abnormal sperm. In the cyst analyzed using the SBEM method, all cells (seven cells were 3D reconstructed) had atypically located mitochondria. The observation of atypically localized mitochondria in all interconnected cells strongly suggests that mitochondrion localization inside the clustered germ cells is governed by factors that act globally in the entire cyst. It is known from numerous studies that clustered cells share macromolecules and other cell components, which are distributed via IBs (Greenbaum et al. 2011; Haglund et al. 2011; Świątek et al. 2014). Thus, the factors governing localization and (fusion/fission?) of mitochondria could also be spread all over the cyst. Another explanation is that the observed atypically located mitochondria are “belated” mitochondria transported to the cytophore along with the residual cytoplasm. However, explaining the lack of mitochondria in the future midpiece is difficult.

The obtained three-dimensional reconstructions allowed us to analyze the spatial conformation of mitochondria. We documented that mitochondria form networks, and individual mitochondria are rare in the case of cysts with spermatogonia and spermatocytes. This condition can be classified as a dynamic hyperfusion using the nomenclature describing mitochondria conformation proposed by Hoitzing et al. (2015), because individual mitochondria are rare and fusion dominates. On the other hand, in the case of cysts with isodiametric, early, and late elongated spermatids, apart from one huge mitochondrion occurring within the cell, few fused mitochondria have been observed in the proximity of the IBs. Maybe there also occurs a dynamic hyperfusion, with a single, elongated mitochondrion continuing the mitochondrial network. It is currently unknown how this large mitochondrion formed. It is most likely that it arose due to the complete fusion of the earlier mitochondrial network. Further analyses are needed to elucidate how the single mitochondrion is formed. In cytophores of cysts with isodiametric, early, and late elongated spermatids, similarly to the previous stages, the mitochondria are in a state of dynamic hyperfusion. Among other Clitellata, the spatial conformation of the mitochondria was analyzed in the sludge worm, *Enchytreus albidus*, in the case of female germline cysts (Urbisz et al. 2020, 2022). Based on numerous methods (including light microscopy, transmission electron microscopy, serial block-face scanning electron microscopy, fluorescence, flow cytometry, and calorimetry), these Authors revealed extensive mitochondrial networks in oocytes, nurse cells, and cytophore. They found that mitochondria are also in a dynamic hyperfusion state, and growing oocytes possess less active mitochondria than the nurse cells (Urbisz et al. 2020, 2022). They confirmed suggestions that the morphology of mitochondria and their distribution depends on the metabolism and the functional state of cells during oogenesis (Urbisz et al. 2020, 2022).

The available literature data show that the energetical activity of mitochondria (i.e., mitochondrial membrane potential) changes during spermatogenesis (Ramalho – Santos et al. 2011; Amaral et al. 2013; Małota et al. 2019). In our studies for analyses of mitochondrial membrane potential, we used the same methods (i.e., JC-1 dye) and parameters as Małota et al. (2019). Our studies showed that the activity of the mitochondria in *H. medicinalis* male germline cysts changes during the successive stages of spermatogenesis. Our results are similar to those published by Małota et al. (2019) regarding the overall changes in mitochondrial activity (measured by the ratio of mitochondria with high and low membrane potential) during the successive stages of spermatogenesis. In the case of *H. medicinalis*, the mitochondrial activity increases in the cells from spermatogonia to the isodiametric spermatid stage and decreases in the following two stages. In the case of *D. veneta* in spermatogonia and isodiametric spermatids, mitochondrial activity is equal, but the activity in spermatocytes is lower compared to these two stages. From isodiametric, early, and late elongated spermatids, mitochondrial activity decreases (Małota et al. 2019). However, in *D. veneta,* the activity of mitochondria in the cytophore is higher in spermatogonia (Małota et al. 2019). Except for this, our results are similar to those obtained in the abovementioned studies. Both in *D. veneta* and *H. medicinalis,* the mean percentage of inactive mitochondria is high; in most cases, over 50%. Małota et al. (2019) suggested that the reason for this situation may be the fact that spermatogenesis is not as an energy-consuming process as it is commonly suggested, or there may be another, yet unknown, reason for their low activity. In the case of medicinal leech cytophore, it was observed that the percentage of inactive mitochondria at all stages of spermatogenesis was maintained at a high level, i.e., over 50%.

The obtained results partially confirmed those previously published. However, our analyses do not directly support the hypothesis of Martinucci et al. (1977) based on studies of male germline cysts in the earthworm *Eisenia fetida*. Martinucci et al. (1977) assumed, due to the high content of mitochondria in the cytophore, that one of the main functions of the cytophore is energy production. Obviously, the change in the activity of mitochondria in germ cells during spermatogenesis is related to processes the cell undergoes during the consecutive stages of spermatogenesis. In the case of spermatogonia, the high mitochondrial potential may be associated with the numerous mitotic divisions (Rooij and Russel 2000; Małota et al. 2019). In spermatocytes, relatively high mitochondrial activity has also been observed, which is associated with another high-energy consuming process, i.e., meiosis (Martínez–Diez et al. 2006; Małota et al. 2019). In *H. medicinalis*, the highest activity of mitochondria was observed in isodiametric spermatids. Most probably, it is related to significant changes within the cell, i.e., chromatin condensation, acrosome, and flagellum formation. However, in early and late elongated spermatids, mitochondria activity is lower than in the previous stage. This finding may indicate that the final stages of spermatogenesis do not require high energy expenditure (Małota et al. 2019).

By correlating the spatial conformation of mitochondria with their activity during the consecutive stages of spermatogenesis, it should be noticed that even though mitochondria are generally connected to a network, their activity fluctuates. Literature data suggest that their activity is higher when mitochondria form a network (Chan 2006b, 2012; Youle and Van Der Bliek 2012). However, in our analyses, we could not compare the mitochondrial activity of the dynamic hyperfusion state with conformations where mitochondria are less fused because extended mitochondrial networks were observed in all analyzed stages. Further analyses concerning the onset of spermatogenesis (e.g., first divisions of spermatogonia) are necessary to see when mitochondria begin forming networks.

## Conclusions

1. In the studied medicinal leeches, the distribution and conformation of mitochondria change during spermatogenesis.
2. Mitochondrial conformation in these cells can be classified as a dynamic hyperfusion.
3. The level of mitochondrial membrane potential in the germline cysts of *H.medicinalis* also changes during spermatogenesis: it grows in cells to the isodiametric spermatids stage and decreases in the next stages. It appears to be related to the processes taking place during spermatogenesis, i.e., cell division and their transformation.
4. Most mitochondria with high MMP occur in cysts with spermatocytes, the lowest activity, measured as low MMP, has been observed in late elongated spermatids.

### Supplementary Information

Below is the link to the electronic supplementary material.

Supplementary information S1. 3D reconstruction of spermatogenic cyst. a) The single cell with cytophore fragment (AVI 8898 KB)

Supplementary information S1. b) The cyst fragment with six cells and cytophore. Yellow – cells; orange – cytophore fragment; light green – nuclei; red – mitochondria; pink – Golgi complex; blue – intercellular bridge (AVI 12335 KB)

Supplementary information S2. 3D reconstruction of spermatocytic cyst. a) The single cell with cytophore fragment (AVI 6742 KB)

Supplementary information S2. b) The cyst fragment with five cells and cytophore. Yellow – cells; orange – cytophore fragment; light green – nuclei; red – mitochondria; pink – Golgi complex; blue – intercellular bridge (AVI 3140 KB)

Supplementary information S3. 3D reconstruction of isodiametric spermatid cyst. a) The single cell with cytophore fragment (AVI 3701 KB)

Supplementary information S3. b) The cyst fragment with five cells and cytophore. Yellow – cells; orange – cytophore fragment; light green – nuclei; red – mitochondria; pink – Golgi complex; blue – intercellular bridge (AVI 3309 KB)

Supplementary information S4. 3D reconstruction of early elongate spermatid cyst. a) The single cell with cytophore fragment (AVI 5989 KB)

Supplementary information S4. b) The cyst fragment with seven cells and cytophore. Yellow – cells; orange – cytophore fragment; light green – nuclei; red – mitochondria; pink – Golgi complex; blue – intercellular bridge (AVI 10856 KB)

Supplementary information S5. 3D reconstruction of late elongate spermatid cyst. a) The single cell with cytophore fragment (AVI 5437 KB)

Supplementary information S5. b) The cyst fragment with nine cells and cytophore. Yellow – cells; orange – cytophore fragment; light green – nuclei; red – mitochondria; pink – Golgi complex; blue – intercellular bridge (AVI 4096 KB)

## Data Availability

The data presented in this study are available on request from the corresponding author.
